# *TaTCP-1*, a Novel Regeneration-Related Gene Involved in the Molecular Regulation of Somatic Embryogenesis in Wheat (*Triticum aestivum* L.)

**DOI:** 10.3389/fpls.2020.01004

**Published:** 2020-09-02

**Authors:** Feifei Li, Xiaoyan Li, Meng Qiao, Bo Li, Dongwei Guo, Xiaohong Zhang, Donghong Min

**Affiliations:** ^1^State Key Laboratory of Crop Stress Biology for Arid Areas and College of Agronomy, Northwest A&F University, Yangling, China; ^2^Crop Research Institute, Shandong Academy of Agricultural Sciences, Jinan, China; ^3^State Key Laboratory of Crop Stress Biology for Arid Areas and College of Life Sciences, Northwest A&F University, Yangling, China

**Keywords:** callus regeneration frequency, gene transformation, somatic embryogenesis, *TaTCP-1*, wheat

## Abstract

The lower regeneration rate of wheat calli is the main factor restricting the development of transgenic wheat plants. Therefore, improving the regeneration rate of wheat callus is a precondition for developing genetic engineering-based wheat breeding approaches. In the present study, we explored the molecular mechanism of wheat regeneration and aimed to establish an efficient system for transgenic wheat. We isolated and identified a regeneration-related gene, *TaTCP-1* (KC808517), from wheat cultivar Lunxuan 987. Sequence analysis revealed that the ORF of *TaTCP-1* was 1623bp long encoding 540 amino acids. The *TaTCP-1* gene was expressed in various wheat tissues. Further, the level of *TaTCP-1* expression was higher in calli and increased gradually with increasing callus induction time, reaching a peak on the 11th day after induction. Moreover, the expression level of *TaTCP-1* was higher in embryogenic calli than in non-embryonic calli. The TaTCP-1 protein was localized to the nucleus, cytoplasm, and cell membrane. The callus regeneration rate of wheat plants transformed with TaTCP-1-RNAi reduced by 85.09%. In contrast, it increased by 14.43% in plants overexpressing *TaTCP-1*. In conclusion, our results showed that *TaTCP-1* played a vital role in promoting wheat regeneration, and regulated the somatic embryogenesis of wheat. These results may have implications in the genetic engineering of wheat for improved wheat production.

## Introduction

With the rapid development of biotechnology, traditional methods, combined with the molecular breeding, are being extensively utilized in wheat breeding. Since the first published report of successful transformation of wheat by microprojectile bombardment using embryogenic calli as explants (Vasil et al., 1992), significant advances have been made in wheat transformation, including the development of different transgenic wheat varieties (Patnaik and Khurana, 2003; Supartana et al., 2005; Ding et al., 2009; Wang et al., 2009), the application of genotype and explant resources (Hess and Carman, 1998; Delporte et al., 2001; He and Lazzeri, 2001; Khurana et al., 2002), and the improvement of selection procedures and the development of co-culture techniques (Janakiraman et al., 2002; Cheng et al., 2003; Khanna and Daggard, 2003; Cheng et al., 2004; Jones et al., 2005; Bhalla et al., 2006). Previous studies focused on increasing the rate of wheat callus regeneration by screening genotypes with higher regeneration potential, using the most effective explants, optimizing the medium composition, and screening of culture conditions. However, the molecular mechanism of callus tissue regeneration remains inadequately analyzed, and therefore, there is a need to understand the molecular mechanism underlying somatic embryogenesis (Boutilier et al., 2002) in wheat.

Somatic embryogenesis is a complex developmental program in which competent somatic cells undergo restructuring through a series of morphological, biochemical, and molecular changes. This requires genome-wide changes in gene expression, which are in turn, regulated *via* epigenetic pathways (Heringer et al., 2013; Li W. et al., 2013; Talapatra et al., 2014; Xu and Huang, 2014; Zhang et al., 2014). Many plant-based studies on regeneration-related gene expression have shown that the molecular changes during somatic embryogenesis involve differential gene expression (Chugh and Khurana, 2002) triggered by a series of signaling cascades. Ikeuchi et al. (2016) have comprehensively summarized the molecular level of plant control regeneration and discussed the influences of efficiency in plant regeneration.

The regeneration ability of plants is related to different genotypes and various types of regeneration. Developmental and environmental constraints influence these regulatory mechanisms (Ikeuchi et al., 2016). Quantitative trait loci (QTL) associated with regeneration-related genes have been identified and mapped in many plant species (Armstrong et al., 1992; Mano et al., 1996; Schiantarelli et al., 2001; Torp et al., 2001; Trujillo-moya et al., 2011; Li S. et al., 2013; Seo et al., 2013). Activation tagging showed that *Sho* was associated with callus regeneration in *Petunia hybrida* L. (Zubko et al., 2002). In a similar study, the cloning of *Os22A* proved its association with the regeneration ability of *Konanso* in rice (*Oryza sativa* L.) (Ozawa and Kawahigashi, 2006). Similarly, *OsNiR* was cloned and proved to be associated with the high regeneration ability of *Kasalath* through Map-based cloning in rice (*Oryza sativa* L.) (Nishimura et al., 2005). Leafy cotyledon 1 (*LEC1*), *LEC2*, and Baby Boom (*BBM*) were reported to be involved in somatic embryogenesis (Zhang et al., 2011). The regeneration in North American Lake Cress, Rorippa aquatica (*Brassicaceae*), was determined, like auxin, gibberellin, and cytokinin, to be important for root regeneration and shoot regeneration (Amano et al., 2020).

The tailless complex polypeptide 1 (TCP-1) protein is a subunit of the hetero-oligomeric chaperonin containing TCP- 1 (CCT). TCP-1 is a member of the chaperonin family that includes GroEL, a 60 kDa heat shock protein (Hsp60), Rubisco subunit binding protein (RBP), and thermophilic factor 55 (TF55) (Kubota et al., 1995). The TCP-1 protein is expressed in all cell types but is abundant in the testis, and was first identified in mice (Ellis, 1992; Nelson and Craig, 1992). Since then, this protein has been identified and characterized in many other animal species, yeast, plants, and protists. The TCP-1 is a component of a hetero-oligomeric 900 kDa double-torus shaped particle with 6-8 different but homologous subunits. It is a highly conserved protein with a molecular weight of approximately 60 kDa (556 to 560 residues). The CCT subunits, beta, gamma, delta, epsilon, zeta, and eta, are evolutionarily related to TCP-1 (Kim et al., 1994; Kubota et al., 1994). In plants, *TCP-1* plays an important role in cytoskeletal organization and cell division. Further, *TCP-1* also acts as a transcriptional regulator, thereby playing an important role in plant growth and regeneration (Feng et al., 2011). TCP proteins were also regulated through growth and developmental processes including branching, floral organ morphogenesis, and leaf growth (İlhan et al., 2018).

Despite being involved in a multitude of processes involved with plant growth and development, the role of *TCP-1* has not been elucidated in wheat regeneration. The present study aimed to establish an efficient regeneration system in wheat by cloning and transferring specific genes involved in the molecular regulation of somatic embryogenesis. The *TaTCP-1* gene was characterized in wheat and its function was analyzed. Further, we analyzed the expression of *TaTCP-1* in transgenic wheat plants to explore its biological role in regeneration.

## Materials and Methods

### Plant Materials

Two winter wheat (*Triticum aestivum* L.) cultivars Lunxuan 987 (with high regeneration potential) and Jimai 22 (with low regeneration efficiency) were used as in this study. The seeds were provided by the State Key Laboratory of Crop Stress Biology for Arid Areas and the College of Agronomy, Northwest A&F University, Yangling, China. The seeds were sown in an experimental field (Northwest A&F University, Yangling, China) in early October of 2012.

### Culture Conditions and Treatments

Fresh intact roots, stems, and, leaves were collected from 3-week-old field-grown wheat. Pistils, stamens, and, glumes were collected at the heading stage. Immature embryos were isolated from seeds 13-14 days after pollination. The immature embryos were sterilized with 70% ethanol for 1 min, dipped in a 20% bleach solution for 15 min, and rinsed three times with the sterilized water. After excising the embryo axis, the immature embryos (1.0-1.2 mm) were removed and inoculated into the SD2 induction medium and cultured for 2 weeks in the dark at 25°C (Tao et al., 2011). The components of the culture medium used in this study are listed in **Table S1**. Induced calli were collected at different stages. All collected samples were immediately frozen in liquid N_2_ and stored at -80 °C.

### *TaTCP-1* Gene Cloning and Sequencing

Regeneration-related candidate gene sequences were analyzed using BLAST. The TCP/cpn60 protein (ACJ54925) from the rice was taken as the query sequence and the wheat EST database (http://www.ncbi.nlm.nih.gov) was searched. The output sequences were assembled by DNAMAN (version 6.0) software. The identified gene was named *TaTCP-1* after identifying the presence of the conserved domain of TCP-1/cpn60.

Total RNA was extracted from 17-day-old calli of cultivar Lunxuan 987 following the manufacturer’s instructions for the EasyPureTM Plant RNA Kit. After treatment with DNase I, a total of 5 µg of RNA was used as a template to synthesize single-strand cDNA using an oligo (dT15) primer following the manufacturer’s protocol (TIAN Script RT Kit, Tiangen, China). The 3’ end of the *TaTCP-1* gene was isolated by using a 3’ Full RACE Core Set Ver.2.0 kit (Takara, Japan) and gene-specific primer GSP *TaTCP* (**Table S2A**). The partial cDNA of *TaTCP-1* was isolated using a 5’ Full RACE kit (Takara, Japan) and gene-specific primers TaTCP-1GSP1 and TaTCP-1GSP2 (**Table S2A**). The full-length cDNA sequence of *TaTCP-1* was amplified by PCR using TaTCP-1F and TaTCP-1R primers and PrimeSTAR^®^ HS DNA Polymerase (Takara, Japan). The PCR products were purified from 1% agarose gel and ligated into the pZero vector (Tiangen, China). The *E. coli* TOP10 competent cells (Tiangen, China) were transformed with the constructed vector by heat shock treatment and identified by sequencing (AuGCT Biological Technology Co., Ltd., China). *TaTCP-1* was cloned from wheat gDNA using gene-specific primers listed in **Table S2A**.

The open reading frame (ORF) of *TaTCP-1* was predicted using the ORF Finder program from NCBI (https://www.ncbi.nlm.nih.gov/orffinder/). Protein molecular weight (MW) and isoelectric point (pI) were calculated by Compute pI/Mw (http://web.expasy.org/compute_pi/). Deduced amino acid sequences of *TaTCP-1* were analyzed by BLAST software. TCP-1 proteins from different species were selected and aligned by DNAMAN 6.0 to construct a phylogenetic tree by the neighbor-joining method using MEGA5 (Tamura et al., 2011).

### Gene Expression Analysis by qRT-PCR

Total RNA was extracted and cDNA was synthesized from embryonic (E) and non*-*embryonic (NE) calli at different stages of induction as previously described. Expression of the *TaTCP-1* gene was analyzed by quantitative real-time PCR (qRT-PCR). The β-actin (GenBank: AB181991) was selected as the endogenous control. The sequences of the primers used for the gene expression analysis are listed in **Table S2B**. PCR amplification was performed using the SuperReal PreMix Plus (SYBR Green) kit (Tiangen, China) following the manufacturer’s instructions. Real-time PCR was carried out on a CFX 96 Real-Time PCR Detection System (Bio-Rad, USA) with the following reaction steps: an initial denaturation at 95°C for 15 min, followed by 40 cycles of amplification (95°C for 10 s, 56°C for 20 s, and 72°C for 30 s) and a final dissociation stage with the temperature increasing from 65°C to 95°C. All reactions were carried out in three independent replicates and the relative gene expression was calculated using the 2^-ΔΔCt^ method (Livak and Schmittgen, 2001).

### Subcellular Localization of *TaTCP-1*

Full-length *TaTCP-1* ORF lacking the termination codon was cloned into a p16318:GFP vector in the sense orientation using the *PstI* and *SalI* restriction enzymes. Protoplasts were prepared from 2-week seedlings according to an earlier reported method by Yoo et al., 2007. The fusion expression vector pGFP-TaTCP-1 and empty control vector p16318:GFP were transferred into prepared protoplasts according to the polyethylene glycol-induced method (Klebe et al., 1983). Transformed protoplasts were cultured in the dark for 16-24 h and gene expression was detected by using a confocal laser-scanning microscope (ZEISS LSM 700; Germany) with an argon laser (488 nm excitation wavelength).

### Heterologous Expression and Purification of *TaTCP-1*

The fusion expression vector pEASY-E1-TaTCP-1 with His-tag was constructed by cloning the *TaTCP-1* gene lacking the termination codon in the pEASY-E1 expression vector (TransGen Biotech Co., Ltd., China). The *E. coli* BL21 (DE3) cells were transformed by the fusion expression vector (pEASY-E1-TaTCP-1) and an empty vector using the heat shock method and cultivated in LB medium containing ampicillin (60 µg/ml). The culture was incubated in a shaker incubator (250 rpm, 37°C) until the OD_600_ reached ~0.5. The expression of the recombinant protein was induced with 0-1 mM β-D-1-thiogalactopyranoside (IPTG) at 37°C for 4, 6, 8, and 10 h. Subsequently, the cells were harvested by centrifugation (7000 rpm) for 3 min and analyzed by SDS-PAGE. The TaTCP-1 protein was purified by ProteinIsoTM Ni-NTA Resin (TransGen Biotech, China) according to the manufacturer’s instructions.

### Overexpression and Silencing of *TaTCP-1* Using Expression Vectors and RNAi

The coding sequences (CDS) of *TaTCP-1* were amplified from the pZero vector and cloned in the expression vector pWMB003 under promoter pUb by using the restriction enzymes SmaI and KpnI. The vector was used to overexpress TaTCP-1 in Jimai 22.

A 303 bp specific cDNA sequence of *TaTCP-1* was used as the interference fragment to construct an RNAi vector. The specific sequence was inserted into the vector pHMW-Adh-Nos in forward and reverse orientations using *SalI*, *BglII*, *EcoRI*, and *BamHI* restriction enzymes to form a hairpin structure (pHMW-Adh-5’F-3’R). The interference fragment was cloned into the expression vector pAHC25 with SmaI and SacI restriction enzymes to construct the RNAi vector pAHC25-TaTCP-1-RNAi. The vector was transferred in Lunxuan 987 to silence the expression of *TaTCP-1*. Herbicide resistance gene (bar gene) was used as a selection marker. The schematic diagram showing the protocol used for vector construction is shown in **Figures S1A**, **B**.

### Transformation, Regeneration, Selection, and Identification of Transgenic Wheat Plants

Immature seeds were collected 13-14 days after pollination from Lunxuan 987 and Jimai 22 wheat and inoculated into the SD2 induction medium. The seeds were cultured for 2 weeks for callus induction. The well-growing calli were transferred into Mo medium for 4-6 h followed by particle bombardment. Empty vectors of pWMB003-TaTCP-1 and pWMB003 were inoculated into Jimai 22, and pAHC25-TaTCP-1-RNAi and pAHC25 empty vectors were inoculated into Lunxuan 987. Following the particle bombardment, the calli were cultured in Mo medium for 16-18 h. The T0 transgenic wheat calli were cultured in the SD2 medium for one week and then transferred to the SD0 medium (auxin free) for another week. The culture process described above was performed in the dark at 25°C. Subsequently, the embryonic calli were transferred into plant regeneration medium and subjected to herbicide selection. The calli were grown at 25 °C with a photoperiod of 16/8 h light/dark. Transgenic plants were transplanted to 25 cm pots and grown in a greenhouse. DNA was extracted from the leaves of the transgenic wheat seedlings to identify plants carrying the specific gene. The transformation procedure is shown in **Figure S2**.

In every generation, the number of inoculated calli, calli with green shoots, green plantlets, and regenerated plants were counted from various types of transformed calli. T3 generation transgenic plants were generated by selection on medium supplemented with herbicide and regenerated by tissue culture technology. T4 immature embryos were used as the explants for further evaluation of the regeneration levels of various types of transgenic wheat plants. One hundred calli from three positive wheat transgene lines were used as the samples for every type of wheat transgene plant. The frequency of embryonic calli was the number of calli with green shoots divided by the total inoculated calli. Regeneration frequency was the number of regenerated plants divided by the total inoculated calli.

### Statistical Analysis

One hundred calli from three positive wheat transgene lines were used as the samples for every type of wheat transgene plant. The data in this study were analyzed using Office 2010 and the variance analysis was performed using SAS software.

## Results

### Cloning and Sequencing of the Wheat Regeneration-Related Gene *TaTCP-1*

**Figure 1:**
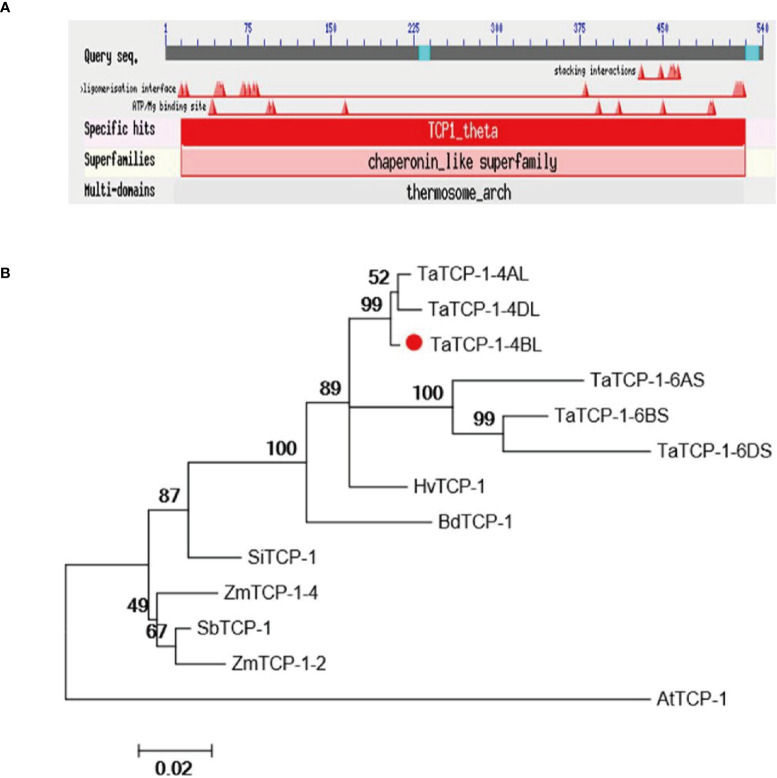
**(A)** The conserved domain and binding site of the TaTCP-1 protein. **(B)** Alignment of protein sequences of TCP-1 from Triticum aestivum, Oryza sativa, Zea mays, Brachypodium distachyon, Sorghum bicolor, Setaria italica, Hordeum vulgare, and Arabidopsis. A neighbor-joining tree (Jones-Taylor-Thornton model) was generated by MEGA6. A bootstrap analysis with 1000 replicates was performed to assess the statistical reliability of the tree topology.

The *TaTCP-1*, a candidate gene (accession number KC808517.1), related to wheat regeneration was isolated from wheat cultivar Lunxuan 987. Sequence analysis indicated the length of cDNA was 2163 bp, while the ORF was 1623 bp long. It encoded 540 amino acids of the TaTCP-1 protein. The full-length gDNA of *TaTCP-1* was 5909 bp with 13 exons and 12 introns. The TaTCP-1 protein contained TCP/cpn60 conserved domain and some special sites, such as ATP binding sites and interaction sites that belonged to the chaperonin-like superfamily (**Figure 1A**). Chaperonins are required for normal cell growth and work as ‘helpers’ for the correct folding and assembly of proteins. The MW of TaTCP-1 was determined to be 58 kDa and the theoretical isoelectric point (pI) was 5.4. The protein showed high homology with HvTCP-1, BdTCP-1, SbTCP-1, SiTCP-1, AtTCP-1, and ZmTCP-1 (**Figure 1B**). The phylogenetic analysis revealed that TaTCP-1 had the highest homology with *Brachypodium distachyon* and *Hordeum* (**Figure S1**).

### Expression Pattern of *TaTCP-1*

**Figure 2:**
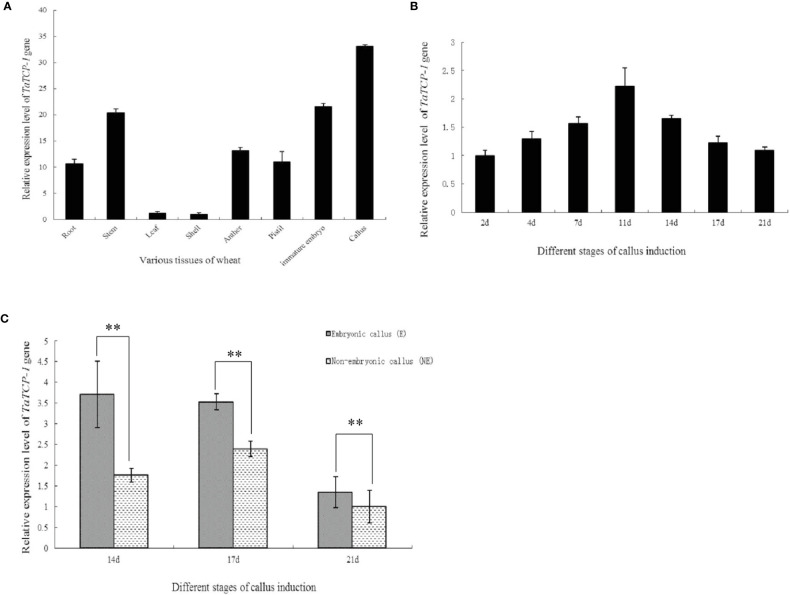
Expression patterns of *TaTCP-1* in eight tissues of wheat **(A)**, different stages of callus induction **(B)**, in embryonic, and non-embryonic calli at different induction stages **(C)**. ** of 0.01 significant.

The expression level of *TaTCP-1* was higher in developing tissues, particularly in actively dividing calli, immature embryos, pistils, and stamens than in glumes and leaves (**Figure 2A**). The expression of *TaTCP-1* was highest in the callus tissue. It was induced in a time-dependent manner (**Figure 2B**) and the expression level reached its highest after 11 days of induction (**Figure 2B**). Significantly, the expression level of *TaTCP-1* was higher in embryonic calli than the non-embryonic calli, which indicated that *TaTCP-1* expression might be associated with wheat regeneration (**Figure 2C**).

### Prokaryotic Expression and Protein Purification of *TaTCP-1*

**Figure 3:**
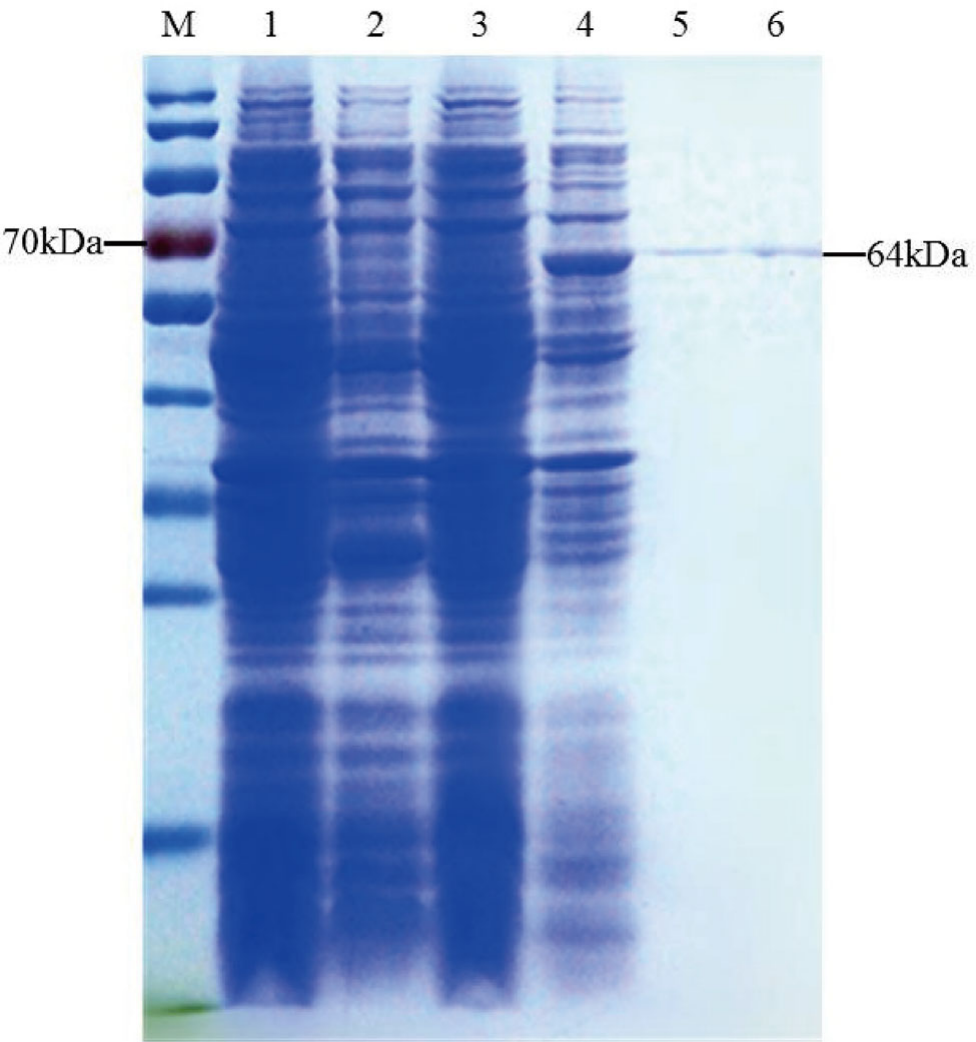
Induction and purification of TaTCP-1. M: Protein ladder; 1: Uninduced pEASY-E1; 2: Induced pEASY-E1; 3: Uninduced pEASY-E1-TaTCP-1; 4: Induced pEASY-E1-TaTCP-1; 5, 6: Purified TaTCP-1.

The recombinant TaTCP-1 protein was ectopically expressed in *E.coli* following transformation with the pEASY-E1-TaTCP-1 and the control plasmid pEASY. Crude lysates were prepared from transformed bacteria following induction and analyzed by SDS-PAGE. The protein band of ~64 kDa, conforming to the theoretical size of TaTCP-1 (58 kDa + His-tag (6 kDa)) (**Figure 3**), was extracted.

### Subcellular Localization of TaTCP-1

**Figure 4:**
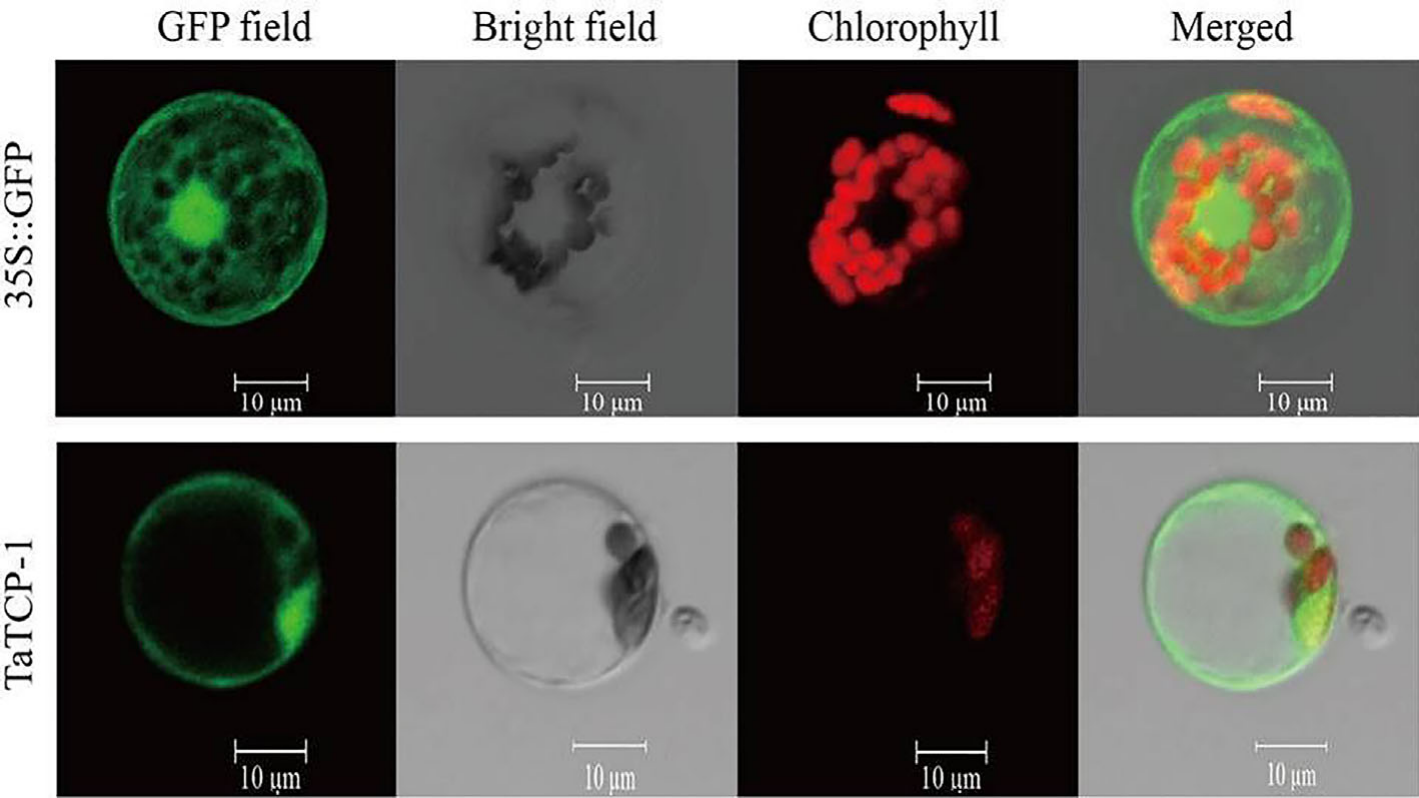
Subcellular localization of TaTCP-1.

Fusion expression vector pGFP-TaTCP-1 and control vector p16318:GFP were transferred to prepared protoplasts to analyze the subcellular localization of the TaTCP-1 protein. Our results showed that the expressed TaTCP-1 was mainly located on the cytomembrane and cell nucleus, and a small amount was present in cytoplasm similar to the free GFP protein (**Figure 4**).

### Identification and Expression of *TaTCP-1* in Transgenic Wheat

**Figure 5:**
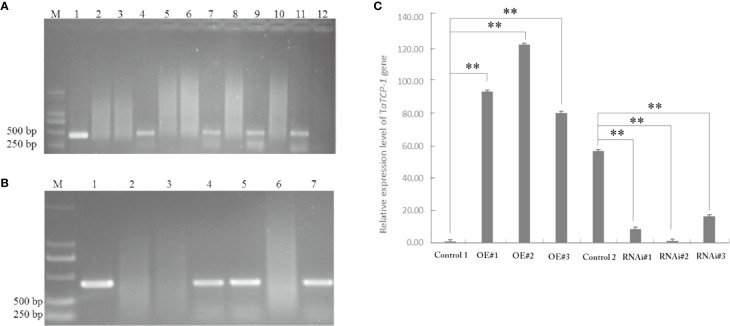
PCR identification of transgenic plants of transgene-overexpressing **(A)** and silenced **(B)**. M: DL2000 DNA marker; 1: Plasmid control; 2: H_2_O control; 3: Wild-type plant; 4-7 or 12: Regenerated plants; **(C)** Comparative analysis of *TaTCP-1* expression by qRT-PCR in transgenic plants. Control 1: Control transgenic wheat plants with the pWMB003 empty vector, OE#1-3: Transgenic wheat plants overexpressing of *TaTCP-1*, Control 2: Control transgenic wheat plants with the pAHC25 empty vector, RNAi#1-3: Transgenic wheat plants in which the expression of *TaTCP-1* gene was silenced. ** of 0.01 significant.

Positive transgenic wheat plants were identified by herbicide selection and genotyping analysis. A total of 720 resistant plants were obtained in T0 including 29 regenerated plants with the PWMB003 empty vector, 146 regenerated plants with the pWMB003-TaTCP-1 vector, 195 regenerated plants with the pAHC25 empty vector, and 350 regenerated plants with the pAHC25-TaTCP-1-RNAi vector (**Table 1**). Results of PCR analysis confirmed 74 positive transgenic wheat plants (**Figures 5A, B**). The transformation rates of wheat plants with the PWMB003 vector, pWMB003-TaTCP-1 vector, pAHC25 vector, and the pAHC25-TaTCP-1-RNAi vector were 0.79%, 0.84%, 1.32%, and 1.21%, respectively (**Table 1**). The expression level of the *TaTCP-1* gene in transgenic plants was analyzed by qRT-PCR (**Figure 5C**). The results from the expression analysis showed that the *TaTCP-1* gene was successfully expressed in Jimai 22 and silenced in Lunxuan 987.

**Table 1:** Statistics analysis of transformation rate and regeneration ability in transgenic wheat.

Transformed plasmid	PWMB003(Control 1)	pWMB003-*TaTCP-1* (OE)	pAHC25(Control 2)	pAHC25-*TaTCP-1* RNAi (RNAi)
No. of bombardment callus	505	2020	1439	2800
No. of T0 regenerate plants	29	146	195	350
No. of T0 positive plants	4	17	19	34
Transformation rate	0.79%	0.84%	1.32%	1.21%
No. of T0 callus	90	408	712	1248
No. of T0 regenerate plants	19	145	1056	789
Regeneration rate of T0	21.11%	35.54%	148.31	63.22%
No. of T1 positive plants	3	8	39	26
No. of T1 callus	85	220	936	702
No. of T1 regenerate plants	18	79	1401	436
Regeneration rate of T1	21.18%	35.91%	149.68%	62.11%
No. of T2 positive plants	3	6	45	28
No. of T2 callus	90	216	942	758
No. of T2 regenerate plants	20	85	1425	452
Regeneration rate of T2	22.22%	39.35%	151.27%	59.63%
No. of T3 positive plants	6	22	625	186

Regeneration rate: Number of resistant regeneration plantlets/Number of calli culturedⅹ100.

**Figure 6:**
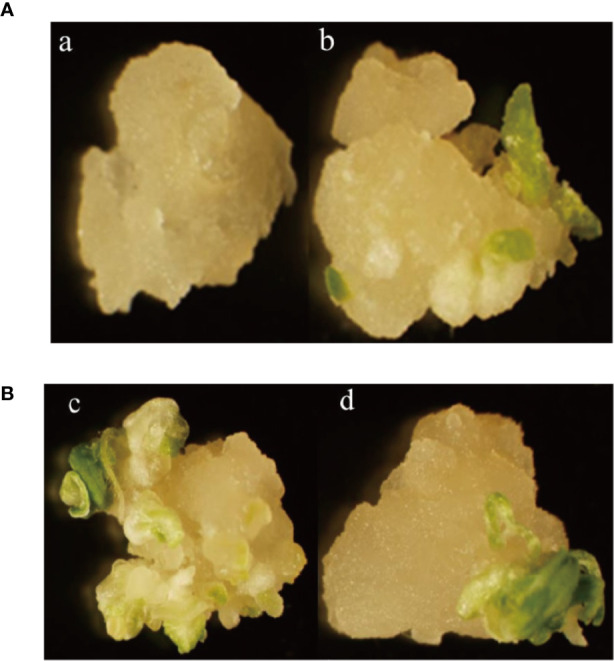
Comparative analysis of the regeneration ability of T3 transgenic wheat lines. **(A)** Comparison between control transgenic wheat plants with the pWMB003 empty vector (a) Transgenic wheat plants of overexpressing *TaTCP-1* gene (b); **(B)** Comparison between control transgenic wheat plants with the pAHC25 empty vector (c) and transgenic wheat plants of silenced *TaTCP-1* gene.

**Figure 7:**
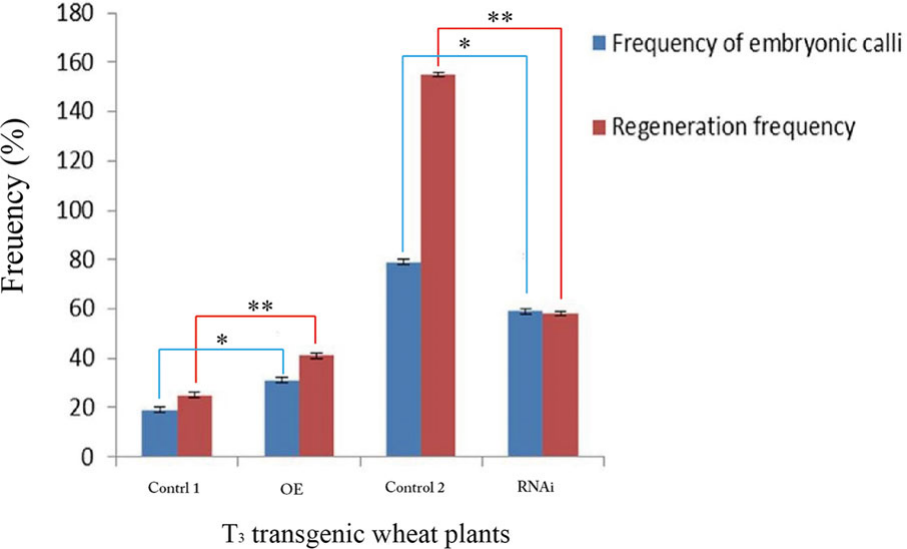
Statistical analysis of the frequency of embryonic calli and regeneration frequency of T3 transgenic wheat lines. Transgenic wheat plants with the pWMB003 empty vector (Control 1), transgenic wheat plants overexpressed *TaTCP-1* gene (OE), control transgenic wheat plants with the pAHC25 empty vector (Control 2) and transgenic wheat plants in which the expression of *TaTCP-1* gene was silenced (RNAi). ** of 0.01 significant and * of 0.05 significant.

The percentage of embryonic calli and regeneration rate of calli were used to evaluate the regeneration level of wheat. After 8 days, the calli reached 2-3 mm^3^ in volume and embryogenic (E) and non-embryogenic (NE) calli were observed. Embryogenic calli were yellow to yellow-green in color and had a smooth or nodular texture. In contrast, the non-embryogenic calli were white in color and had limpid, watery, and friable structures (**Figure 6**). In T3 transgenic wheat overexpressing *TaTCP-1* over-expression (OE), the percentage of embryonic calli and callus regeneration efficiency increased by 12% and 18% compared to the wheat transformed with the pWMB003 empty vector (control 1) (**Table 2**). After 25 days of culture in regeneration medium, embryonic calli of green spots were visible in approximately 79% of calli in the control transgenic wheat plants transformed with the pAHC25 empty vector; however, these spots were visible in only 59% of calli from transgenic wheat plants transformed with *TaTCP-1* silencing RNAi (**Table 3**). Further, the frequency of embryonic callus induction and plantlet regeneration decreased significantly (by 20% and 97%, respectively) in transgenic wheat transformed with *TaTCP-1* RNAi when compared to the wheat transformed with the pAHC25 empty vector (control 2) (**Table 3**). Moreover, the ability of embryogenic callus induction and regeneration of immature embryos in the transgene-overexpressing and silenced plants showed a statistically significant difference when compared to plants transformed with control (**Figure 7** and **Table S3**). Significantly, wheat transformed with TaTCP-1-RNAi cassette showed negative effects on regeneration efficiency, while *TaTCP-1* overexpression had positive effects on regeneration efficiency. Collectively, these results indicated that the *TaTCP-1* gene plays a vital role in promoting wheat regeneration.

**Table 2:** The comparative analysis of regeneration ability in T3 transgenic wheat plants.

Transgenic wheat plants	No. of calli cultured	Frequency of embryonic calli (%)^1)^	Regeneration frequency (%)^2)^
Control 1	100	19	25
OE	100	31	43
CK2	100	79	155
RNAi	100	59	58

1) Number of embryonic calli/Number of calli cultured ⅹ 100.

2) Number of resistant regeneration plantlets/Number of calli cultured ⅹ 100.

**Table 3:** The comparative analysis of regeneration ability in T0, T1, T2, and T3.

Transformed plasmid	PWMB003 (Control 1) (%)	pWMB003-*TaTCP-1* (OE) (%)	pAHC25 (Control 2) (%)	pAHC25-*TaTCP-1*RNAi (RNAi) (%)
Regeneration rate of T0	21.11	35.54	148.31	63.22
Regeneration rate of T1	21.18	35.91	149.68	62.11
Regeneration rate of T2	22.22	39.35	151.27	59.63
Regeneration rate of T3	25	43	155	58

## Discussion

Earlier studies have shown that the explant type and genotype, hormonal and sugar composition of medium, induction by sugar alcohols (mannitol or sorbitol), the number of steps, and explant exposure times during a particular stage were the main factors to influence successful culture of plant cells and tissues *in vitro* (Li et al., 2004; Vunsh et al., 2007). The regenerative ability of different plants depends on different genotypes (Bregitzer, 1992; Filippov et al., 2006). Therefore, identification of regeneration-related genes is an important step in the development of effective methods for facilitating plant regeneration. Genes such as *SERK* have been successfully cloned from *Arabidopsis thaliana* (Banno et al., 2001; Hecht et al., 2001), rice (Hu et al., 2005), wheat (Singla et al., 2008; Yakandawala and Jordan, 2008), rye (Gruszczynska and Rakoczy-Trojanowska, 2011), and maize (Zhang et al., 2011). The *TCP-1* was proved to play an important role in plant growth and regeneration (Feng et al., 2011). In the present study, we cloned the *TaTCP-1* gene in common wheat and performed its functional analysis to elucidate its role in wheat plant regeneration. The *TaTCP-1* gene is located on the long arm of the 4B chromosome (4BL) of the wheat genome. We identified five highly homologous *TaTCP-1*-encoding genes in wheat that were localized on 4AL, 4DL, 6AS, 6BS, and 6DS chromosomes and had 90% homology (**Figure 1B**). Our results showed that the *TaTCP-1* gene was mainly expressed in actively dividing tissues and organs. Significantly, the expression pattern of the *TaTCP-1* was different from another regeneration-related gene, *NiR*, reported earlier (Nishimura et al., 2005).

The regeneration rate of most T3 generation transgenic plants was higher than that of the T1 and T2 generation plants except those transformed with the RNAi transgene. The higher regeneration rate of T3 transgenic plants may be caused by better regulatory conditions. Our results showed that the regeneration rate of transgenic wheat plants, transformed with the *TaTCP-1-*RNAi, decreased with increasing generations, indicating that the genotype played a more important role than the environmental conditions on the regeneration ability of wheat. Moreover, the influence of *TaTCP-1* silencing was more obvious on the plant regeneration than its overexpression. This could be explained by the complexity of the wheat genome. There are six highly homologous *TaTCP-1*-related genes in wheat, and therefore, transformation with the *TaTCP-1*-RNAi may not only silence the *TaTCP-1* gene on 4BL but may also downregulate related genes. This may explain the pronounced effects observed in plants transformed with the *TaTCP-1-*RNAi than those transformed to overexpress the *TaTCP-1*.

In summary, in the present study, we isolated *TaTCP-1*, a novel gene related to regeneration, and demonstrated its ability to influence the high regeneration abilities of transgenic explants. The *TaTCP-1* gene was also proved to play a key role in the molecular regulation of the somatic embryogenesis of wheat. Collectively, our results suggest that *TaTCP-1* could be used as a novel selection marker to identify transformed wheat. It offers the advantage of avoiding the use of antibiotics and herbicide selection, thereby reducing the possibility of resistance development in such plants. Therefore, our results not only suggest the possibilities of advancing wheat transgenic technology but also facilitate the construction of a safe transgenic wheat system.

## Data Availability Statement

The raw data supporting the conclusions of this article will be made available by the authors, without undue reservation, to any qualified researcher.

## Author Contributions

XZ and DM conceived the project, FL and MQ conducted the experiments, and FL, XL, MQ, BL, and DG performed the data analysis. FL and XL wrote the manuscript. All authors contributed to the article and approved the submitted version.

## Funding

This work was supported by research grants from the National Transgenic Key Project of the Ministry of Agriculture of China (2016ZX08002002-010), Molecular Design Breeding of Wheat of National Key R&D Program of China (2016YFD0101802), Programme of Introducing Talents of Innovative Discipline to Universities (Project 111) from the State Administration of Foreign Experts Affairs (#B18042) “Crop breeding for disease resistance and genetic improvement”, Tang Scholar of Northwest Agriculture and Forestry University.

## Conflict of Interest

The authors declare that the research was conducted in the absence of any commercial or financial relationships that could be construed as a potential conflict of interest.

## Abbreviations

Bp, Base pair; BLAST, Basic local alignment search tool; Cdna, Complementary DNA; GFP, Green Fluorescent protein; Amp, Ampicillin; LB, Luria-Bertani medium; mRNA, Messenger RNA; NCBI, National Center for Biotechnology Information; PCR, Polymerase Chain Reaction; RT-PCR, Reverse Transcription Polymerase Chain Reaction; qRT-PCR, Quantitative Real-time Polymerase Chain Reaction.
