# Supplementary material for: TaTCP-1, a Novel Regeneration-Related Gene Involved in the Molecular Regulation of Somatic Embryogenesis in Wheat (Triticum aestivum L.)
Source: Front Plant Sci. 2020 Sep 2;11:1004. doi: 10.3389/fpls.2020.01004 (PMC7492748; doi:10.3389/fpls.2020.01004)
Supplement: Supplementary file 1 [file DataSheet_1.zip › Supplementary Tables.docx]

**Table S1.** Nutrient medium used in this study

|  | SD2 | SD0 | Mo | B2 or B4 | SRN | ZMM |
| --- | --- | --- | --- | --- | --- | --- |
| MS mineral salt（10×）/ml  MS mineral salt（500×）/ml  Fe（200×）/ml  2,4-D（2mg/mL）/ml  VB1（1mg/mL）/ml  Mannitol /g  sorbitol/g  IAA（1mg/mL）/ml  NAA（0.1mg/mL）/ml  Met（1mg/mL）/ml  MS organics (100×)/ml  B2 (200×)/ml  Bialaphos（2mg/mL）/ml  sucrose/g  aspartic acid+/g  phytagel/g  agar /g | 100  2  5  1  1  —  —  —  —  —  —  —  —  30  0.15  2.4  — | 100  2  5  —  1  —  —  —  —  —  —  —  —  30  0.15  2.4  — | 100  2  5  1  1  36.5  36.5  —  —  —  —  —  —  30  0.15  2.4  — | 50  1  2.5  —  —  —  —  —  —  —  —  5  1 or 2  20  —  2.4  — | 50  2  5  —  —  —  —  —  1  0.5  10  —  —  30  —  —  8 | 50  2  5  —  —  —  —  1  1  3  10  —  —  30  —  —  8 |

| primer name | primer sequence（5’- 3’） | purpose |
| --- | --- | --- |
| TaTCP-1 F  TaTCP-1 R  5’RACE GSP Outer Primer F  5’RACE GSP Outer Primer R  5’RACE GSP Inner Primer F  5’RACE GSP Inner Primer R  3’RACE GSP Outer Primer F  3’RACE GSP Outer Primer R  3’RACE GSP Inner Primer F  3’RACE GSP Inner Primer R  A-F  D-R  C-F  F-R  F-F  I-R  I-F  K-R  K-F  M-R | CTCTCACCGCTGCCGAC  TGCTTGGGTTTCATGGTTGG  CATGGCTACATGCTGACAGCCTA  CCTCAAGCTCGTTCACGATTGT  CGCGGATCCACAGCCTACTGATGATCAGTCGATG  GATGTTCTTCAGGACGGCCTCGT  CCTCTATGTTACAAAGTCCTTCGCT  TACCGTCGTTCCACTAGTGATTT  CTTGTTTCCTGTGTATCATGCTGTGG  CGCGGATCCTCCACTAGTGATTTCACTATAGG  ATGGTGGGATACGGGAT  CTCCAACCAGCTTTGCCAC  GGATGTCAGGAACAGGGAGG  CCAACTGCTGCTCCACTGAC  GGAAGCAAAGGTGGAGGAGC  CACCATCATCGACAGCTCTTTC  GGTTGGATCAGTATGCTATTGC  GCAGCAGCACCAGGGATAAT  CGGACTTGGTGCAATGGAG  CTAGTCTTCATCAATGCCA | RT-PCR  RT-PCR  RACE  RACE  RACE  RACE  RACE  RACE  RACE  RACE  Amplification of gDNA  Amplification of gDNA  Amplification of gDNA  Amplification of gDNA  Amplification of gDNA  Amplification of gDNA  Amplification of gDNA  Amplification of gDNA  Amplification of gDNA  Amplification of gDNA |

**Table S2-a.** Primers used for cloning of *TaTCP-1*

**Table S2-b.** Primers used for function analysis of *TaTCP-1*

| Name | Primer sequence（5’- 3’） | Purpose |
| --- | --- | --- |
| S-F  S-R  Actin F  Actin R  G-F  G-R  Ubi-F  Nos-R  Z-F  Z-R  F-F  F-R  T-F  Nos-R  Bar-F  Bar-R | GGTTGGATCAGTATGCTATTGC  CATCCTCGCAAGCACCT  CTCCCTCACAACAACCGC  TACCAGGAACTTCCATACCAAC  **TCCCCCGGG**ATGGTGGGATACGGGATC(*Sma*Ⅰ)  **GGGGTACC**CTAGTCTTCATCAATGCCACCT(*Kpn*Ⅰ)  TCGATGCTCACCCTGTTGTTTG  GCAAGACCGGCAACAGGATTC  **ATGTCGACCCCGGG**GGTGAAAGTTTTGAAATGGTTCC（*Sal*Ⅰ+*Sma*Ⅰ）  **CATAGATCT**GTCTTCATCAATGCCACCTTG（*Bgl*Ⅱ）  **ATAGAATTCGAGCTC**GGTGAAAGTTTTGAAATGGTTCC(*EcoR*Ⅰ+*Sac*Ⅰ)  **TATGGATCC**GTCTTCATCAATGCCACCTTG (*BamH*Ⅰ)  GGTTCCAAGAACATTGTCTG  GCAAGACCGGCAACAGGATTC  CTGCACCATCGTCAACCACTACATC  AGCTGCCAGAAACCCACGTCAT | qRT-PCR  qRT-PCR  qRT-PCR  qRT-PCR  Construction of pWMB003-TaTCP-1  Construction of pWMB003-TaTCP-1  Detection of pWMB003-TaTCP-1  Detection of pWMB003-TaTCP-1  Construction of pAHC25-TaTCP-1RNAi  Construction of pAHC25-TaTCP-1RNAi  Construction of pAHC25-TaTCP-1RNAi  Construction of pAHC25-TaTCP-1RNAi  transgenic detection  transgenic detection  transgenic detection  transgenic detection |

**Table S3. The original statistical data of each strain (Contro1, OE, Contro2 and RNAi).**

| Transgenic wheat plants | No. of calli cultured | No. of embryonic calli | Frequency of embryonic calli(%)^1)^ | Resistant regeneration plantlets | Regeneration frequency(%)^2)^ |
| --- | --- | --- | --- | --- | --- |
| Control 1 # 1 | 35 | 6 | 17.14 | 9 | 25.71 |
| Control 1 # 2 | 33 | 8 | 24.24 | 8 | 24.24 |
| Control 1 # 3 | 32 | 5 | 15.63 | 8 | 25 |
| OE # 1 | 34 | 11 | 32.35 | 16 | 47.06 |
| OE # 2 | 34 | 10 | 29.41 | 14 | 41.18 |
| OE # 3 | 32 | 10 | 31.25 | 13 | 40.63 |
| Control 2 # 1 | 35 | 28 | 80 | 54 | 154.29 |
| Control 2 # 2 | 32 | 25 | 78.13 | 50 | 156.25 |
| Control 2 # 3 | 33 | 26 | 78.79 | 51 | 154.55 |
| RNAi # 1 | 35 | 22 | 62.86 | 21 | 60 |
| RNAi # 2 | 31 | 15 | 48.39 | 13 | 41.94 |
| RNAi # 3 | 34 | 22 | 64.71 | 24 | 70.51 |
