# Supplementary figures and images for: TaTCP-1, a Novel Regeneration-Related Gene Involved in the Molecular Regulation of Somatic Embryogenesis in Wheat (Triticum aestivum L.)
Source: Front Plant Sci. 2020 Sep 2;11:1004. doi: 10.3389/fpls.2020.01004 (PMC7492748; doi:10.3389/fpls.2020.01004)

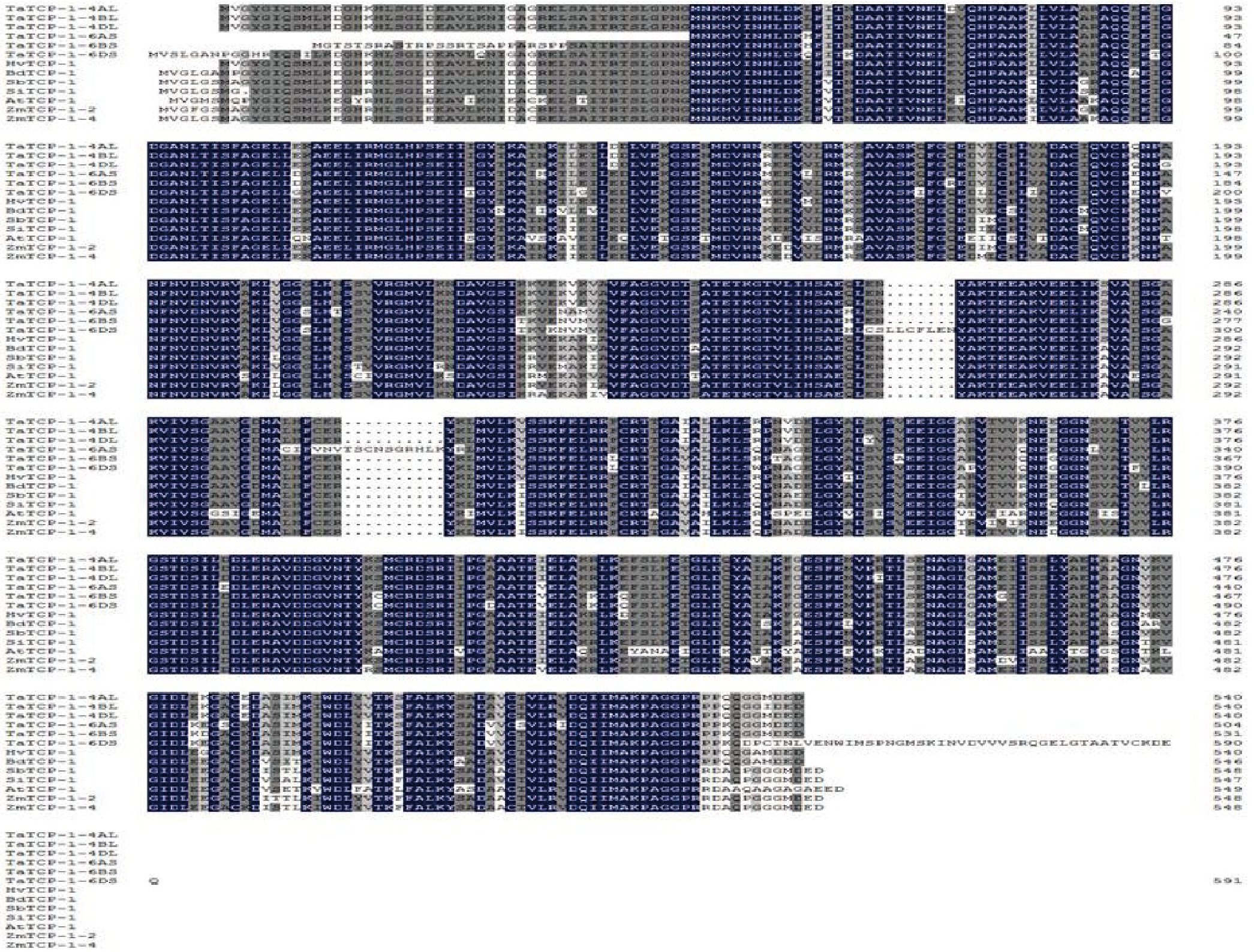

Supplement: Supplementary file 1 [file DataSheet_1.zip › Figure S1.tif]

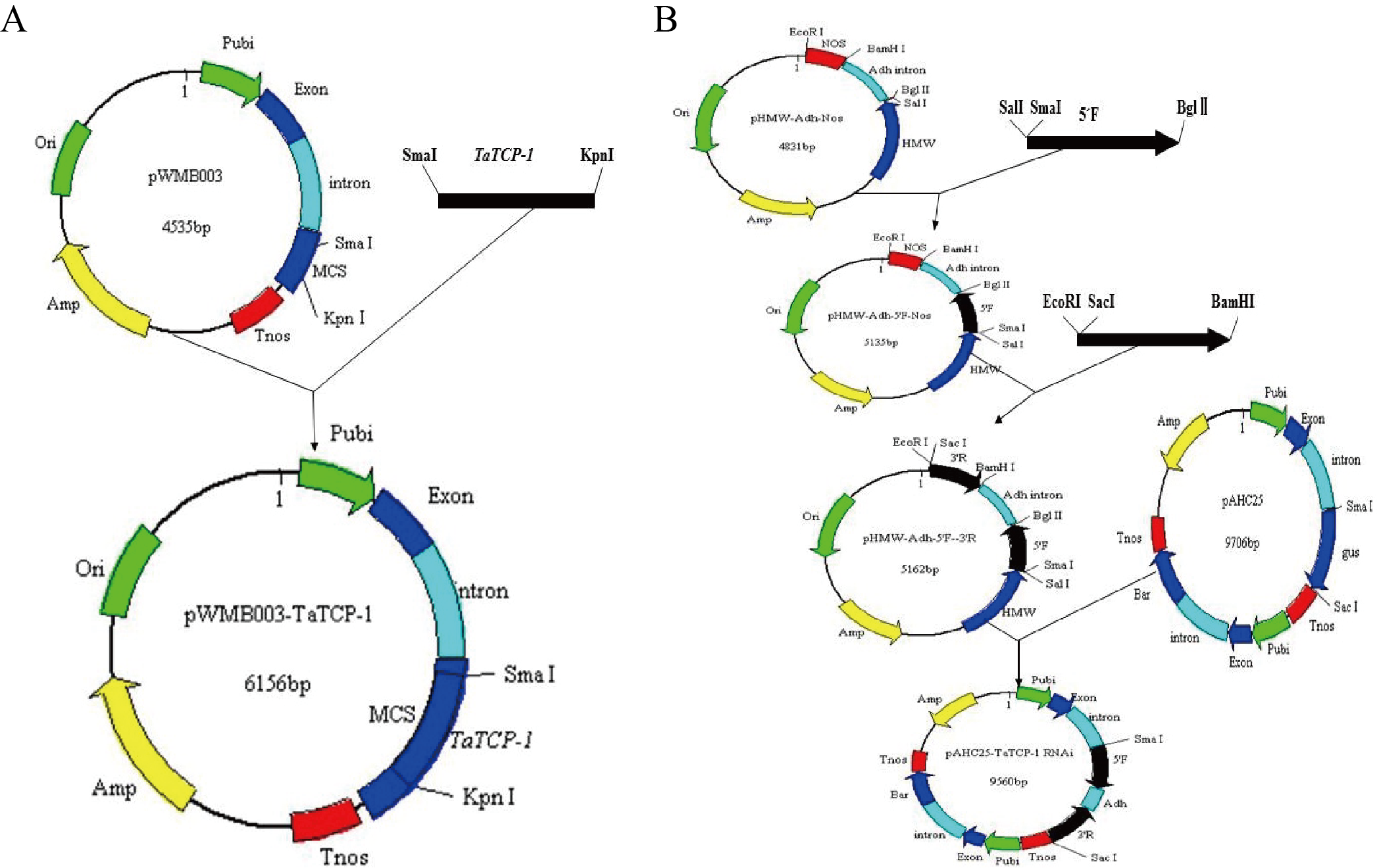

Supplement: Supplementary file 1 [file DataSheet_1.zip › Figure S2.tif]

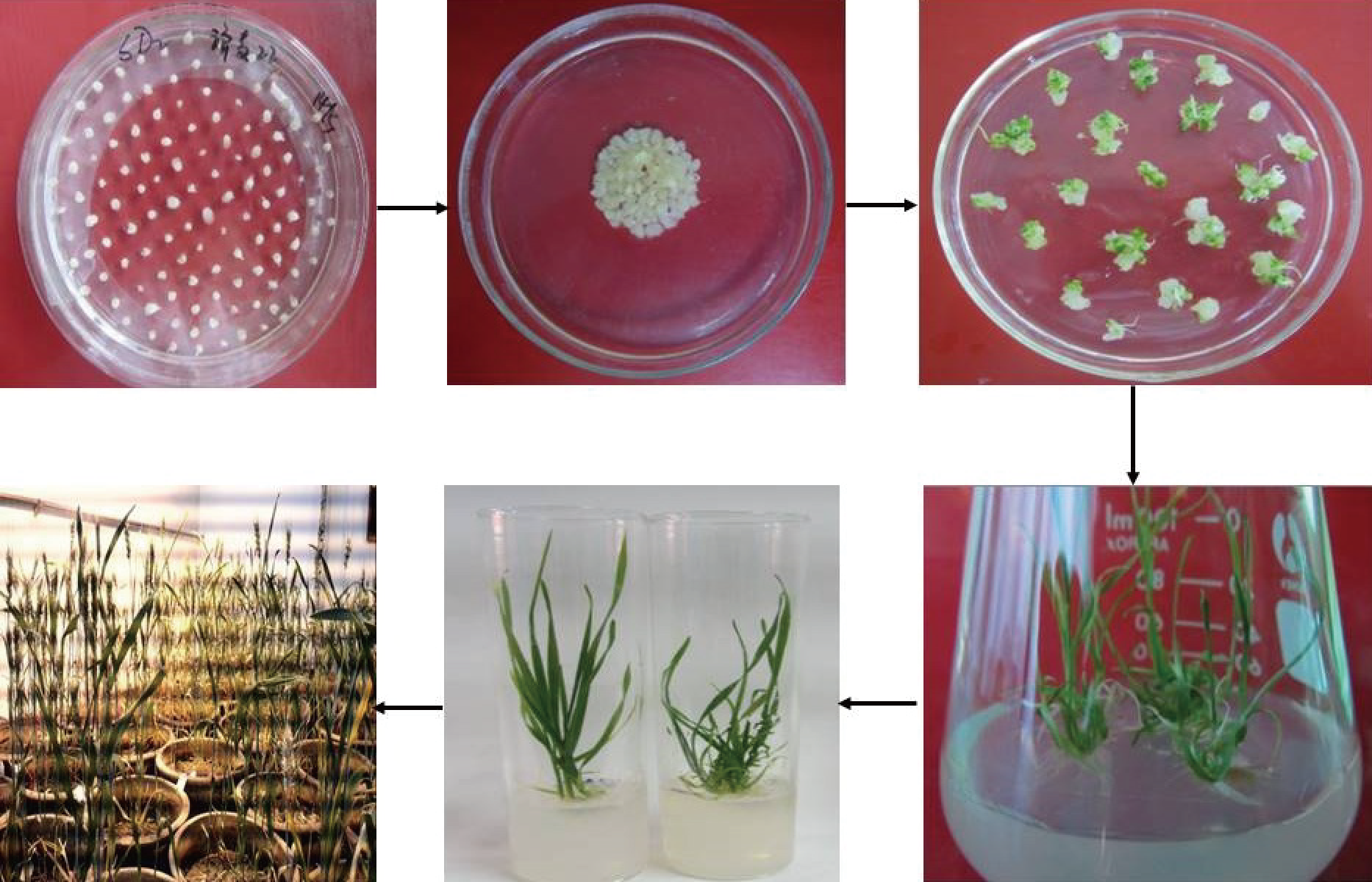

Supplement: Supplementary file 1 [file DataSheet_1.zip › Figure S3.tif]
